# A Comparative Study of Geometric Phase Change- and Sideband Peak Count-Based Techniques for Monitoring Damage Growth and Material Nonlinearity

**DOI:** 10.3390/s24206552

**Published:** 2024-10-11

**Authors:** Guangdong Zhang, Tribikram Kundu, Pierre A. Deymier, Keith Runge

**Affiliations:** 1 New Frontiers of Sound Science and Technology Center, University of Arizona, Tucson, AZ 85721, USA; guangdongzhang@arizona.edu (G.Z.); deymier@arizona.edu (P.A.D.); krunge@arizona.edu (K.R.); 2 Department of Aerospace and Mechanical Engineering, University of Arizona, Tucson, AZ 85721, USA; 3 Department of Civil and Architectural Engineering and Mechanics, University of Arizona, Tucson, AZ 85721, USA; 4 Department of Materials Science and Engineering, University of Arizona, Tucson, AZ 85721, USA

**Keywords:** topological acoustic sensing, geometric phase change-index (GPC-I), sideband peak count-index (SPC-I), damage monitoring, material nonlinearity, numerical modeling, structural health monitoring

## Abstract

This work presents numerical modeling-based investigations for detecting and monitoring damage growth and material nonlinearity in plate structures using topological acoustic (TA) and sideband peak count (SPC)-based sensing techniques. The nonlinear ultrasonic SPC-based technique (SPC-index or SPC-I) has shown its effectiveness in monitoring damage growth affecting various engineering materials. However, the new acoustic parameter, “geometric phase change (GPC)” and GPC-index (or GPC-I), derived from the TA sensing technique adopted for monitoring damage growth or material nonlinearity has not been reported yet. The damage growth modeling is carried out by the peri-ultrasound technique to simulate nonlinear interactions between elastic waves and damages (cracks). For damage growth with a purely linear response and for the nonlinearity arising from only the nonlinear stress–strain relationship of the material, the numerical analysis is conducted by the finite element method (FEM) in the Abaqus/CAE 2021 software. In both numerical modeling scenarios, the SPC- and GPC-based techniques are adopted to capture and compare those responses. The computed results show that, from a purely linear scattering response in FEM modeling, the GPC-I can effectively detect the existence of damage but cannot monitor damage growth since the linear scattering differences are small when crack thickness increases. The SPC-I does not show any change when a nonlinear response is not generated. However, the nonlinear response from the damage growth can be efficiently modeled by the nonlocal peri-ultrasound technique. Both the GPC-I and SPC-I techniques can clearly show the damage evolution process if the frequencies are properly chosen. This investigation also shows that the GPC-I indicator has the capability to distinguish nonlinear materials from linear materials while the SPC-I is found to be more effective in distinguishing between different types of nonlinear materials. This work can reveal the mechanism of GPC-I for capturing linear and nonlinear responses, and thus can provide guidance in structural health monitoring (SHM).

## 1. Introduction

Ultrasonic nondestructive testing and evaluation (UNDT&E)-based techniques are widely used in engineering structural health monitoring (SHM) to ensure structural safety [1,2]. Nonlinear ultrasonic (NLU) techniques, due to their high sensitivity, are receiving increasing attention over conventional linear acoustic techniques [3,4,5]. Robust techniques with high sensitivity are desirable for the development of reliable SHM techniques.

In general, monitoring damage or material evolution using ultrasonic waves relies on linear or nonlinear changes in the waves’ main characteristics such as velocity, amplitude, resonance frequency and dynamic phase [6]. For instance, at the early stages of acoustics research, linear parameters like the wave velocity, attenuation and dynamic phase changes were commonly used to detect damage features such as material evolution, porosity of additive manufacturing parts and cracks in plate structures [7,8,9,10]. In recent decades, some classic NLU techniques, such as higher harmonics generation (HHG) [11,12,13] and nonlinear wave modulation spectroscopy (NWMS) or frequency modulation (FM) [14,15] have been used to detect changes in material states. In nonlinear resonance techniques, such as nonlinear impact resonance acoustic spectroscopy or NIRAS, resonance frequency shifts and attenuation variations with increasing amplitudes of excitation are recorded and analyzed [16,17]. Recently, another nonlinear technique called a sideband peak count-index (or SPC-I), which focuses on changes in multiple sideband peak amplitudes, has been proposed [18,19,20,21,22,23,24,25]. A correlation between the amplitude levels of sideband peaks and the degree of nonlinear response has been established [26]. It can be concluded that most currently available NLU techniques are focused on variations in nonlinear amplitude and frequency and can be referred to as magnitude-based or frequency feature-based methods. In spite of the high sensitivity of NLU techniques, limitations and restrictions still exist in terms of their practical implementation.

An emerging method called topological acoustic (TA) sensing has recently been introduced to sense defects and environmental and structural changes [6,27,28,29,30,31]. This method utilizes a geometric phase change (GPC) that quantifies the variation in the geometric phase of an acoustic field represented as a state vector in Hilbert space. The states of the acoustic field in the unperturbed (damage-free) and perturbed (damaged) states are mapped as multidimensional vectors in the Hilbert space and compared. By exploiting sharp topological features spanned by the acoustic field’s multidimensional state vector, the geometric phase sensing modality can achieve higher sensitivity than magnitude- or frequency-based sensing approaches. This approach is not limited to linear wave fields but can also include nonlinear contributions to the wave field and their associated state vectors.

With the TA sensing technique, changes in complex environments such as forests [27] or the state of permafrost in the arctic [28] have been monitored using seismic waves. This method has been further extended to monitor perturbations in the form of (1) a mass defects located on an array of coupled acoustic waveguides [6], (2) mass defects in a nonlinear granular metamaterial [29] and (3) a small subwavelength object on a flat surface submerged in water [30]. These investigations reported in the literature have introduced the capability of GPC to sense defects by analyzing linear scattering. Such sensitivity with GPC is useful in damage detection. Zhang et al. [31] adopted the GPC technique to monitor damage growth in heterogeneous structures with different topographies using nonlocal peri-ultrasound modeling. They found that GPC is superior to the amplitude-based SPC technique for monitoring damage growth in heterogeneous structures. In peri-ultrasound modeling, both linear and nonlinear scattering can be generated by cracks. Separate investigations of linear and nonlinear responses are needed to arrive at a clear understanding of the mechanism of GPC and its capability of sensing and monitoring damage. Investigations of monitoring material nonlinearity using GPC are also very few.

In this work, in light of the above discussion, a TA sensing technique using GPC is investigated numerically in terms of detecting and monitoring damage and material nonlinearity in plate structures. The GPC-index (GPC-I) is used to detect and monitor perturbations (such as defects) and material evolution using the TA sensing technique. The numerical modeling is carried out by nonlocal peri-ultrasound modeling of damage growth that produces both linear and nonlinear responses. The finite element method (FEM) in the Abaqus/CAE 2021 software is used for damage growth modeling that produces pure linear responses. The comparison of these two numerical methods can thus provide a comprehensive understanding of the effectiveness of the GPC technique for detecting and monitoring damage growth in structures using its linear and nonlinear responses. The material nonlinearity is modeled in FEM by considering different stress–strain relationships prescribed by the Abaqus/CAE 2021 software. The effect of different constitutive material relationships on the GPC-I and SPC-I techniques are also investigated and compared.

Therefore, the five unique features and original contributions of this paper are:

(1) The GPC-I is proposed for the first time as a damage indicator with high sensitivity;
(2) The effects of linear and nonlinear responses on the behavior of GPC-I are investigated in depth;
(3) A comparison between the available SPC-I technique and the proposed GPC-I technique can reveal the mechanism GPC-I uses to capture linear and nonlinear responses, and thus can provide guidance for SHM applications;
(4) The effects of material nonlinearity on SPC-I and GPC-I are compared;
(5) A fundamental understanding of geometric phase change in practical applications is clarified.

## 2. Methodology and Theory

In this section, the concept and methodology of the proposed TA sensing techniques are presented, the GPC plots are generated and the GPC-index (GPC-I) parameter is defined. The crack-induced nonlinear mechanism in the peri-ultrasound modeling framework [32,33,34] and the effect of material nonlinearity on elastic wave propagation [18,19] have already been reported on in the literature and are not repeated here.

**Figure 1:**
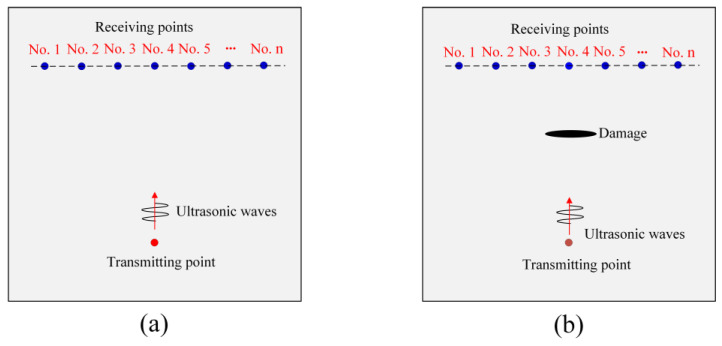
Schematic diagram showing the reference state and perturbed state for TA sensing: (**a**) damage-free plate (reference state); (**b**) damaged plate (perturbed state).

First, we consider the acoustic fields in plate structures without and with damage to illustrate the process of topological acoustic sensing. The schematic diagram is shown in Figure 1. It should be noted that a TA sensing method can be applied to any complex geometry. Here, a simple plate structure is taken as an example to describe the methodology.

For the reference state (intact plate without any damage) at each receiving location (total *n* locations) shown in Figure 1a, the displacement or velocity values are recorded as a time series. Each of these *n* time series are fast Fourier transformed (FFT) to obtain complex amplitudes in the spectral domain. At a given frequency, these *n* complex amplitudes can be represented as a normalized state vector in a n dimensional complex Hilbert space. The *n* basis vectors of that subspace correspond to locations in the physical space. This normalized state vector can be written as [30]

(1) $$C=\frac{1}{\sqrt{C_{1}^{2}+C_{2}^{2}+C_{3}^{2}+\ldots C_{n}^{2}}}\left(\begin{array}{c} C_{1}e^{i\phi_{1}}\\ C_{2}e^{i\phi_{2}}\\ C_{3}e^{i\phi_{3}}\\ \ldots \\ C_{n}e^{i\phi_{n}} \end{array}\right)$$

In Equation (1), $C_{k}$ and $\phi_{k}$ (*k* = 1, 2, 3, …, *n*) are the magnitude and spatial phase at each receiving point. The components of this multi-dimensional state vector are the complex amplitudes of the field at every location in the discretized space of the *n* detectors. When damage is introduced, as shown in Figure 1b, the perturbation in the physical space scatters the acoustic waves and modifies the spatial distribution of the acoustic field. Perturbations such as damage then change the normalized complex amplitude of the acoustic field to

(2) $$C\prime =\frac{1}{\sqrt{C_{1}^{\prime 2}+C_{2}^{\prime 2}+C_{3}^{\prime 2}+\ldots C_{n}^{\prime 2}}}\left(\begin{array}{c} C_{1}^{\prime }e^{i\phi_{1}^{\prime }}\\ C_{2}^{\prime }e^{i\phi_{2}^{\prime }}\\ C_{3}^{\prime }e^{i\phi_{3}^{\prime }}\\ \ldots \\ C_{n}^{\prime }e^{i\phi_{n}^{\prime }} \end{array}\right)$$

At a single given frequency *f*, the angle between the vector representation of the acoustic field along the *n* locations in the damage-free and damaged systems corresponds to a change in the geometric phase of the acoustic wave. This angle or single geometric phase change at the given frequency *f* can be obtained through the dot product of these two state vectors and can be expressed as

(3) Δφ=arcosRe⁡C*·C′,      Δφ∈0,π

where C* denotes the complex conjugate of state vector C while Re stands for the real part of a complex quantity.

**Figure 2:**
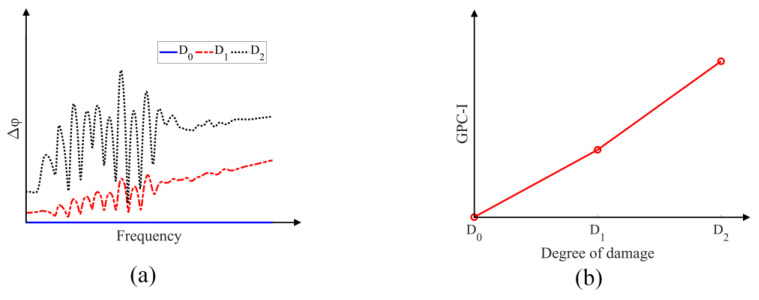
(**a**) Example of GPC plots; (**b**) GPC index (GPC-I) values for damage-free and damaged structures.

Generally, the acoustic signals at each receiving point contain multiple frequencies because input excitation signals own the bandwidth which contains multiple frequency components, then a series of geometric phase changes at each frequency component can be plotted as a function of frequency, as shown in Figure 2a, with different levels of damage (D_0_—no damage, D_1_—medium level of damage and D_2_—higher level of damage). The spectral dependency of the GPC Δφ measures changes in the spatial characteristics of the acoustic field during the wave propagation due to perturbations—the larger the perturbations are in structures the higher the GPC values are in the plots. The GPC-index (GPC-I) is defined as the average value of each GPC curve in the frequency-dependent plots. The GPC-I values for different degrees of damage in Figure 2a are shown in Figure 2b.

From Figure 2, it is clear that the damage levels in plate structures can be related to the acoustic parameter GPC-I. In this work, the effects of linear scattering response and the nonlinear response due to damage growth on the behavior of GPC-I and SPC-I are numerically investigated. The capabilities of GPC-I and SPC-I for sensing material nonlinearity (not considering the linear scattering by the damage) is also examined.

## 3. Model Description and Numerical Modeling

### 3.1. Peri-Ultrasound and FEM Modeling for Cracked Geometry

**Figure 3:**
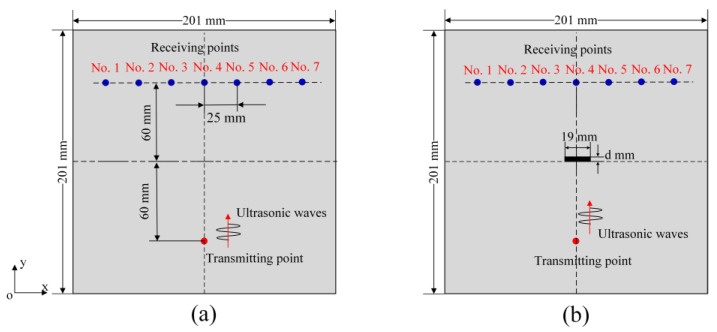
Two-dimensional view of the problem geometry for TA sensing: (**a**) crack-free plate (reference state); (**b**) cracked plate (perturbed state).

As illustrated in Section 2, for TA sensing the vector representation of an acoustic field in an infinite dimensional Hilbert space can be compared for damage-free and damaged plates. However, for practical applications, a much smaller discretized n-dimensional subspace is considered to describe the acoustic field. In this investigation, this subspace is generated from seven receiving points (so *n* = 7) as shown in Figure 3. It should be noted that in TA sensing at least two receiving points are needed to reflect the spatial characteristics of the acoustic field. More receiving points improve the spatial resolution of the acoustic field and its geometry. Here, seven receiving points are distributed symmetrically about the *y*-axis, as shown in Figure 3a (without any cracks) and in Figure 3b (with one crack). The distance between two adjacent receiving points is set at 25 mm. The dimensions of the plate structure for numerical modeling are $201\times 201\times 3{\mathrm{mm}}^{3}$. The thickness d of the crack in Figure 3b takes values 0, 1, 2 and 4 mm for modeling damage growth in the plate and the length of the crack is fixed at 19 mm. A plate having no cracks is considered as the reference state or reference shape with respect to which the cracked plates (perturbed states) are compared. For wave propagation modeling, the vertical distances from the transmitting point and the seven receiving points to the *x*-axis are set at 60 mm. The crack is symmetrically placed about the *y*-axis with the bottom surface coinciding with the *x*-axis and the crack thickness increases as the top surface moves in the positive y direction. The material properties considered for the numerical modeling are listed in Table 1.

**Figure 4:**
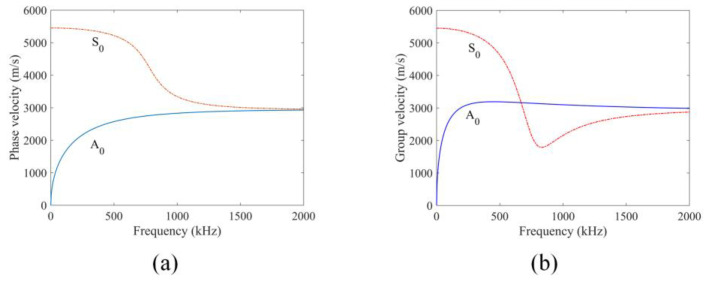
Dispersion curves for a 3 mm thick aluminum plate: (**a**) Phase velocity; (**b**) group velocity.

To arrive at a better understanding of how elastic waves propagate in the plate considered here, the phase velocity dispersion curves of a 3 mm thick aluminum plate are computed using the material properties given in Table 1. Figure 4 shows the plots of phase and group velocity dispersion curves of the S_0_ and A_0_ guided wave modes in the plate.

In the mesh-free peri-ultrasound modeling, the entire plate structure is discretized into cubes with side length of 1 mm. Therefore, 201 cubes are taken in the x direction for the problem geometry shown in Figure 3. The horizon size defined in peri-ultrasound modeling is selected as δ=3.015Δx following references [32,33,34] to ensure both computational efficiency and accuracy, where Δx is selected as 1 mm, which denotes the side length of the cube. All output variables are recorded on particles at receiving locations distributed symmetrically about the *y*-axis. Cracks are formed by removing one or more layers of cubes from the plate structure. Note that for each removed layer in the y direction, 19 cubes are removed in the x direction and 3 such layers are removed in the z direction to form the through-thickness crack. It should be emphasized that in the nonlocal peri-ultrasound modeling, the cracks are modeled by removing material cubes and thus the overall stiffness of the region near the crack is also reduced. The nonlinearity arising from the interactions between the propagating elastic waves and cracks is captured indirectly through the interactions between the material points located on the two sides of a crack when the points are located within a horizon despite being separated by the crack. In the purely linear elastic continuum model, elastic waves cannot pass through any crack of a finite thickness. When the crack thickness increases, the interactions between the points on the two sides of the crack decrease. In this manner the effect of the clapping phenomenon is indirectly captured since the clapping of thin cracks also allows more wave energy to pass through a thin crack compared to a thick crack. Thus, both peri-ultrasound modeling and the crack clapping phenomenon which is also referred as contact acoustic nonlinearity (CAN) [35,36,37,38] result in more interactions between the points on the two sides of the crack for thin cracks compared to thick cracks [39]. Cracks can be classified as “thin crack” or “thick crack” depending on the horizon radius mentioned in nonlocal peridynamics theory. Here, in our peri-ultrasound modeling, the horizon size is 3.015 mm (with a 1 mm side length of the cube) so cracks with a thickness of 0 mm, 1 mm and 2 mm are thin cracks and crack with a thickness of 4 mm is a thick crack. In FEM modeling, cracks are simply formed by removing materials from the pristine plate and the whole plate is meshed using the Abaqus/CAE software.

For both FEM and peri-ultrasound modeling, a Hanning window modulated excitation displacement field with a central frequency of 200 kHz and with two cycles is applied at the transmitting point to excite the plate structure in the negative z direction. For wave propagation with FEM modeling, the whole plate structure is discretized into finite elements. To ensure the computation and accuracy of the numerical model, it is essential to determine and adopt the appropriate element size (*L*) and the integration time step (Δt). These two parameters can be determined from Equations (4) and (5) [40]

(4) $$L\leq \frac{\lambda_{\min}}{10}$$

(5) $$\Delta t\leq \frac{1}{20f_{\max}}$$

For the central frequency of 200 kHz, the minimum wavelength ($\lambda_{\min}$) for the A_0_ mode is 10.035 mm according to the dispersion curves, and the time step is 0.25 μs. The grid size is selected as 1 mm to ensure that whole plate structures are divided into an integer number of cells. The time step is selected as 0.02 μs so the sampling frequency for recording the signals is 50 MSa/s (mega samples per second) to ensure computation accuracy.

At the receiving points, velocity values (in the z direction or perpendicular to the plate surface) for each crack thickness are recorded at every calculation step to obtain the time history signal at each receiving point. Thus, seven signals are recorded at seven receiving points for every crack thickness: 0 mm (no crack), 1 mm, 2 mm and 4 mm. Then, GPC analysis is carried out with these signals.

### 3.2. FEM Modeling for Material Nonlinearity Problems

**Figure 5:**
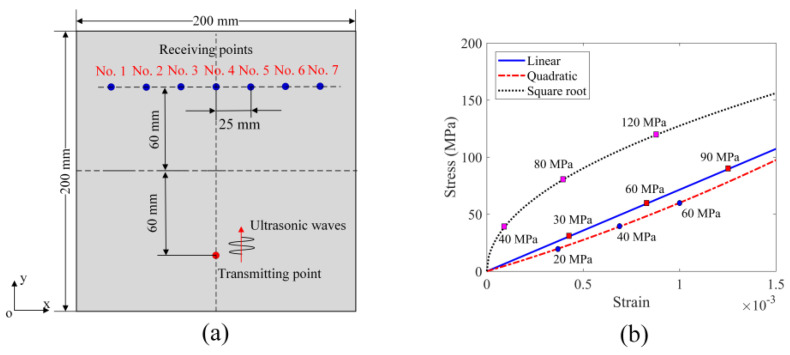
Two-dimensional view of the problem geometry of the plate structure and stress–strain relationships for wave propagation modeling: (**a**) problem geometry; (**b**) stress-stain relations.

The effect of material nonlinearity on GPC is investigated by FEM modeling. The 2-D view (the xy plane) of the problem geometry that is modeled is shown in Figure 5a. The dimensions of the plate structure are $200\times 200\times 3{\mathrm{mm}}^{3}$, the linear elastic material properties for numerical modeling are listed in Table 1 and the nonlinear material properties (stress–strain relationships) are prescribed in the Abaqus/CAE software. For wave propagation setup, the excitation point or the transmitting point is selected on the top surface of the plate, on the *y*-axis, and the seven receiving points are distributed symmetrically about the *y*-axis. The vertical distance of the horizontal central axis of the plate from the transmitting point as well as the seven receiving points is 60 mm as shown in the figure.

Five different stress–strain relationships are considered for the numerical investigation with FEM modeling. Figure 5b shows three of the five stress–strain relationships that are considered. The middle solid line indicates a linear stress–strain relationship where the stiffness remains constant; the lower dashed line gives a quadratic relationship between stress and strain where the stiffness increases as the load increases; the top dotted line shows a square root stress–strain relationship where the stiffness decreases as the load increases. The three numbers marked on each curve prescribe the stress amplitude when the material is loaded along the assumed stress–strain curve. Two additional stress–strain relationships that are also considered but not shown in the figure correspond to stress being proportional to (i) the cubic of strain and (ii) the one-third root of strain. The amplification factor (AF) is defined as the ratio of the excitation amplitude to a reference amplitude (the reference value is the minimum value, making AF always greater than 1) [26].

The 3-D plate structure is discretized into a number of finite elements in the Abaqus/CAE 2021 software. Every element has a side length of 1 mm. Such discretization produces 201 nodes along the *x*-axis, thus making a transmitting point located on the central line (*y*-axis). Receiving points and other nodes are distributed symmetrically about the *y*-axis. This is consistent with what is achieved in the peri-ultrasound modeling described in Section 3.1. An explicit dynamic solution scheme is adopted for our calculation. The initial excitation is applied to the transmitting point in the negative z direction, and the velocity values in the z direction, perpendicular to the plate surface, are recorded at the seven receiving points for geometric phase analysis. The sampling frequency at the receiving point is 50 MSa/s (mega samples per second). For each stress–strain relationship shown in Figure 5b, there are three recorded signals which correspond to the AF values equal to 1, 2 and 3, respectively, at each receiving point, so a total of nine signals are recorded at each receiving point for the material nonlinearity analysis by the TA sensing technique.

## 4. Numerical Modeling Results

### 4.1. Effects of FEM and Peri-Ultrasound Modeling on Wave Propagation

Both local FEM modeling and nonlocal peridynamics (PD)-based peri-ultrasound modeling are used to investigate the effect of crack initiation and growth on the wave propagation behavior and GPC calculation. In FEM modeling, the surfaces of cracks remain separated as the waves propagate, so elastic waves cannot pass through an open crack but are scattered by it. However, in nonlocal peri-ultrasound modeling, due to the nonlocal effect, waves can pass through a crack, so both linear and nonlinear responses are generated.

**Figure 6:**
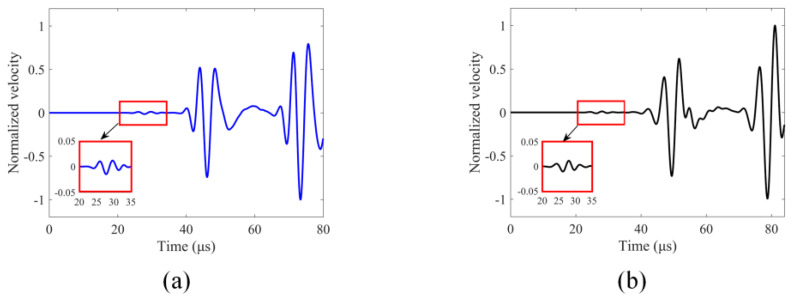
Normalized velocity time history at receiver 4 for no-crack case (0 mm thick crack) generated by: (**a**) finite element method (FEM); (**b**) peridynamics (PD)-based peri-ultrasound modeling technique.

In the TA sensing technique the GPC parameter can theoretically sense both linear and nonlinear responses. However, separate as well as combined investigations of linear and nonlinear effects on GPC can provide guidance on the applicability of GPC for sensing micro-cracks (nonlinear responses). A comparison of the results of these two modeling techniques in terms of the geometric phase change (GPC) can help us to understand the effects of linear and nonlinear responses separately. First, for the no-crack case, we compare the results generated by these two numerical methods for wave propagation modeling. Normalized velocity values received at receiver 4 are shown in Figure 6.

**Figure 7:**
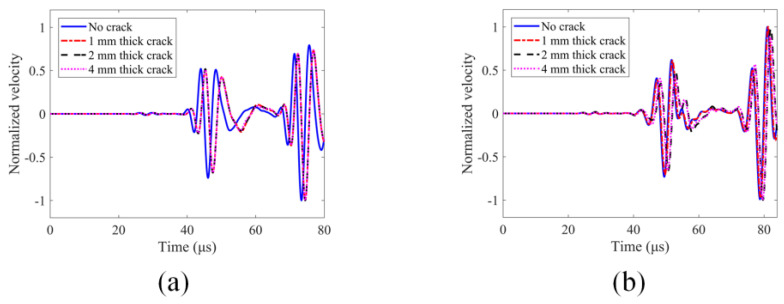
Normalized velocity time history at receiver 4 for four cases (0 mm or no-crack, 1 mm, 2 mm and 4 mm thick cracks) from two different numerical modeling techniques: (**a**) finite element method (FEM); (**b**) peridynamics (PD)-based peri-ultrasound modeling.

For the no-crack case, it can be seen from Figure 6 that the two numerical modeling techniques generate similar results but are, strictly speaking, not identical since one modeling technique is based on the nonlocal theory while the other one is not. However, both numerical modeling methods clearly show different guided wave modes where they are expected to appear in the time history plots according to the dispersion curves shown in Figure 4. Hence, we can have confidence in our computed results. The normalized velocity values at receiver 4 for the four crack thicknesses (0 mm, 1 mm, 2 mm and 4 mm) generated by these two numerical modeling techniques are also compared and shown in Figure 7.

**Figure 8:**
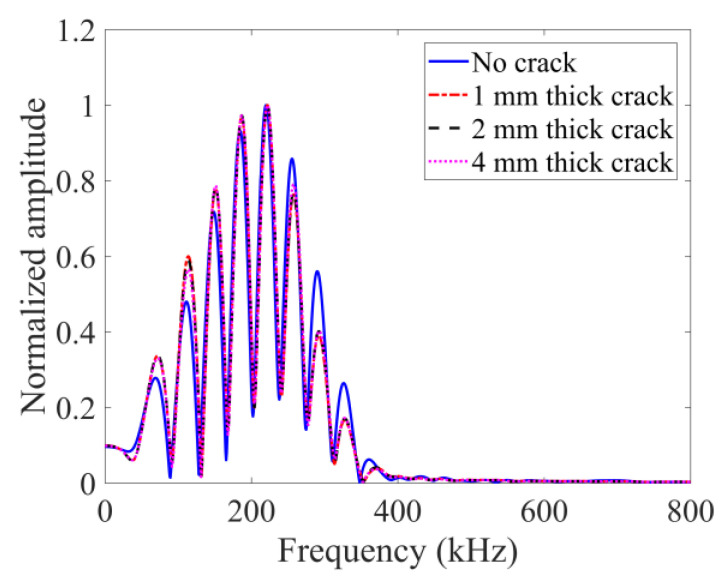
Normalized spectral plots at receiver 4 from FEM modeling technique for no-crack case and three different thicknesses of crack.

**Figure 9:**
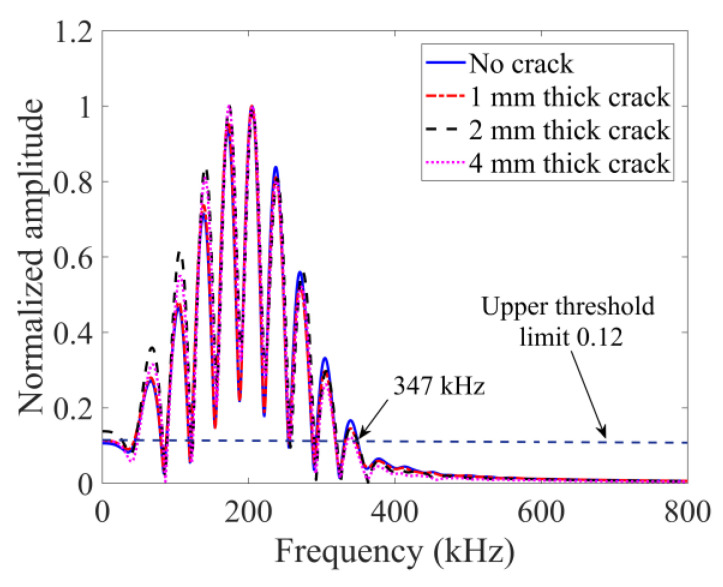
Normalized spectral plots at sensor 4 from PD-based modeling technique for no-crack and three different thicknesses of cracks.

In the FEM modeling results shown in Figure 7a, where only linear scattering is generated from the cracks, there are significant changes between the no-crack case (0 mm thick crack) and the cracked cases (1 mm, 2 mm and 4 mm). However, there are almost no changes for 1 mm, 2 mm and 4 mm thick cracks (these three curves overlap). Therefore, it can be concluded that damage growth (the increase in crack thickness) does not significantly affect the pure linear scattering response. However, for the peri-ultrasound modeling results shown in Figure 7b, where elastic waves can pass through the no-crack case as well as through some cracks (having 1 mm and 2 mm thicknesses) and to a lesser degree through the 4 mm thick crack [32,33], both linear and nonlinear responses are generated. The linear response is from the reflection, refraction and scattering of the waves by the crack’s surface while the nonlinear response is from the nonlocal effects when some elastic wave energy passes through the cracks. In contrast to FEM modeling, in peri-ultrasound modeling there are significant changes in time histories for 1 mm, 2 mm and 4 mm thick cracks. Since we have pointed out and concluded that in FEM modeling a purely linear scattering response does not change when crack thickness increases, the changes observed due to crack thickness variations in the peri-ultrasound modeling results must be due to the nonlinear effects generated by the cracks. The effect of damage growth (the increase in crack thickness) on spectral plots is shown in Figure 8 and Figure 9. The multiple peaks and dips shown in the spectral plots are generated by the constructive and destructive interferences between the incident wave from the source and the reflected wave from the boundary at the sensor location. In the time histories shown in Figure 7, both incident and reflected waves appear and thus affect the spectral plot shown in Figure 8. The spectral plots clearly show the distortions and frequency shifts as the crack thickness increases from 0 to 4 mm.

For the purely linear scattering response in FEM shown in Figure 8, there is almost no change on the spectral envelope shape for the four cases, indicating that no additional frequency components are generated. Also, there are no differences for the three crack thicknesses (1, 2 and 4 mm). This clearly proves that the crack thickness increase does not affect the purely linear scattering from cracks.

In the peri-ultrasound modeling results shown in Figure 9, it can be observed that the envelopes for 2 mm and 4 mm thick cracks are distorted (the 1 mm thick crack case is also slightly distorted compared to the no-crack case). Therefore, additional frequency components are generated. Such additional frequency components indicate the presence of nonlinearity due to cracks.

The nonlinear behaviors from the cracks are also analyzed with the help of the sideband peak count-index (or SPC-I) parameter. We took the upper threshold limit (horizontal dashed line) to be 0.12 as shown in Figure 9. The last intersection point of this horizontal line with the spectral plots is located approximately at the 347 kHz frequency value. The sideband peaks located beyond this frequency contribute to the SPC-I calculation. This frequency boundary is also adopted in TA sensing to calculate GPC-I for a comparison of these two techniques (SPC-I and GPC-I).

### 4.2. GPC Computation for Damage Growth

**Figure 10:**
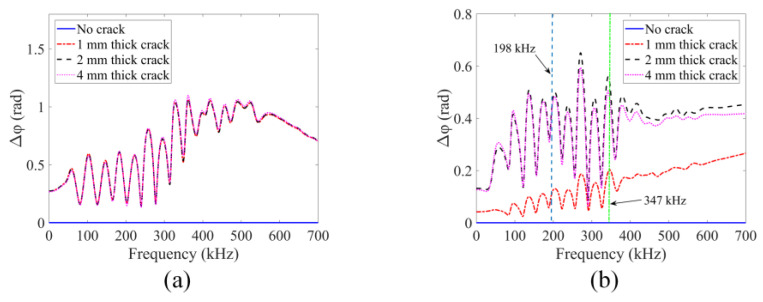
Crack effects on GPC calculation obtained from two different numerical modeling techniques: (**a**) FEM modeling; (**b**) peri-ultrasound modeling.

In this section, GPC which is from the TA sensing methodology mentioned in Section 2 is analyzed. From the z direction velocity spectral plots recorded by seven receivers, the GPC plots generated by the two numerical modeling techniques are obtained and shown in Figure 10.

**Figure 11:**
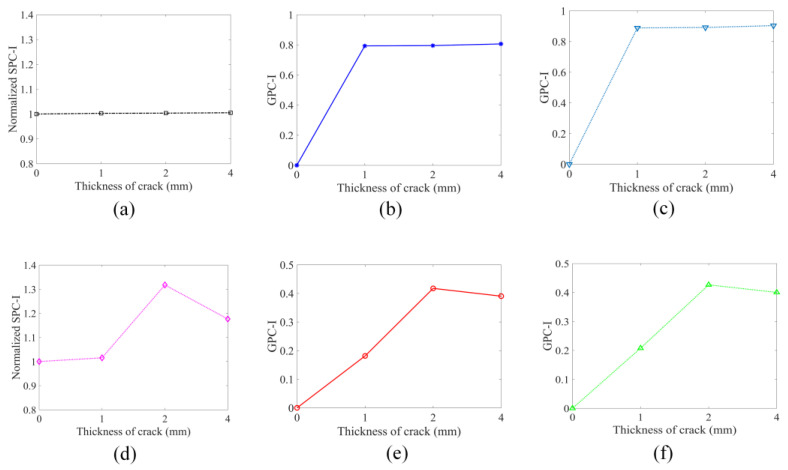
Variations in the crack thickness effects on SPC-I (the first column) and GPC-I (the second and third columns). The second column is calculated by taking the frequency range from 198 kHz to 700 kHz, and the third column is calculated in the frequency range of 347 kHz to 700 kHz for proper comparison with the SPC-I values. The FEM (the top row) and the peri-ultrasound (the bottom row) results capture the linear response and the combined linear and nonlinear responses, respectively.

For the purely linear scattering response, it can be seen from Figure 10a that the frequency-dependent GPC is sensitive to the crack appearing in the structure—multiple sharp peaks and dips can be observed for the cracked plates. However, no measurable changes are noticed when crack thickness increases from 1 mm to 4 mm. Therefore, the linear scattering response of GPC is not sensitive to damage growth. In the peri-ultrasound modeling results shown in Figure 10b, both linear and nonlinear responses are caused by the cracks. Clear differences are observed when crack thickness increases (indicating damage growth). At the higher frequency ranges beginning from about 198 kHz (the first vertical dashed line in Figure 10b), the GPC plots begin to show a trend which is different from the trend observed in the lower frequency range (below 100 kHz). At lower frequencies, the peak values in Figure 10b increase with crack thickness but at higher frequencies (above 198 kHz) the peak values first increase for crack thicknesses up to 2 mm and then decrease. The higher frequency trend is consistent with previous investigations with SPC-I [18,38,39,40] where it was shown that the nonlinear parameter SPC-I value first increases and then decreases with damage growth. Since the SPC-I technique mainly captures the nonlinear response caused by the damage, it can be considered to be a nonlinearity monitoring technique. The reason for this initial increase followed by a decreasing trend is that when cracks are at their early stages (micro-cracks) the two surfaces of the cracks come into contact as the waves propagate through them, causing contact acoustic nonlinearity (CAN) and the SPC-I value increases. When these micro-cracks coalesce to form macro-cracks, the crack surfaces do not come into contact to generate nonlinearity and the SPC-I value decreases. Therefore, GPC can capture a nonlinear response due to damage growth when it is calculated at the appropriate frequency. It should be noted that at higher frequencies the wavelength is smaller. When the crack thickness increases from 2 mm to 4 mm, the nonlinear response decreases for smaller wavelengths. This is because for smaller wavelengths the two sides of the cracks do not interact to generate the nonlinear effect but at lower frequencies with larger wavelengths they do. The normalized SPC-I variations with an upper threshold limit of 12% (as stated earlier) are obtained from signals at receiver 4 using these two numerical modeling techniques. The SPC-I variations are shown in Figure 11a,d (the first column in Figure 11).

The geometric phase change-index (or GPC-I) is defined as the average value of whole GPC plots or partial GPC plots over certain frequency ranges depending on which parts of each GPC plot are our main interest. Figure 10b gives the frequency range for calculating the GPC-I values. At higher frequency ranges, from around 198 kHz, the GPC trends are consistent with the SPC-I variation trend. The frequency range from 198 kHz (the first vertical dashed line in Figure 10b) to 700 kHz is considered to obtain GPC-I values from the two numerical modeling techniques, and the GPC-I variations are shown in Figure 11b,e (the second column). For proper comparison with the SPC-I results, a second frequency range from 347 kHz (the second vertical line in Figure 10b) to 700 kHz is considered to obtain GPC-I values from the two numerical modeling techniques. The GPC-I variations obtained from this analysis are shown in Figure 11c,f (the third column).

It can be seen from the FEM modeling results (the top row in Figure 11), that the normalized SPC-I value which mainly captures the nonlinear response in structures shows almost no variation in Figure 11a, confirming that in FEM modeling only a linear scattering response is generated. The GPC-I results can detect the formation of a crack (or damage) but cannot monitor crack growth since in Figure 11b,c almost a horizontal line is obtained as the crack thickness changes. In the peri-ultrasound modeling results (the bottom row in Figure 11), both GPC-I and SPC-I show consistent trends—first increasing up to 2 mm and then decreasing, thus forming a ‘hump’. In several experimental and theoretical investigations, this trend in SPC-I variation—first increasing and then decreasing, forming a hump—has been reported [18,41,42]. It gives us confidence in the numerical modeling results presented in this paper. In practical applications with real structures, such a hump indicates that the nonlinearity is reaching a maximum value because of the highest density of micro-crack accumulation and then the nonlinearity starts to decrease as the micro-cracks start to coalesce to form macro-cracks. Thus, such a hump in both GPC-I and SPC-I plots can serve as a warning sign for macro-crack formation, indicating that the damage growth has reached a critical value. In our modeling, thicker cracks represent macro-cracks while thinner cracks are representative of micro-cracks since the acoustic energy can pass through the thinner cracks but not through the thicker cracks.

### 4.3. GPC Computation for Material Nonlinearity

**Figure 12:**
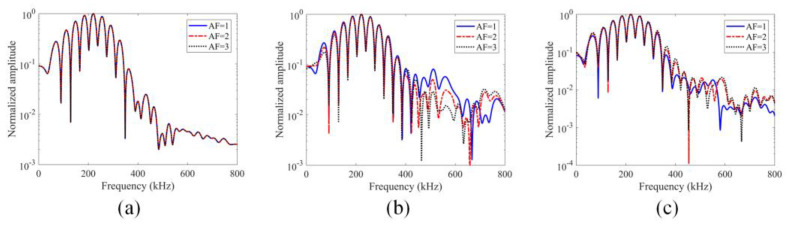
Normalized spectral plots for material nonlinearity for different stress–strain relationships: (**a**) linear stress–strain relationship; (**b**) quadratic stress–strain relationship; (**c**) square root stress–strain relationship.

The investigations of the effect of material nonlinearity on GPC were carried out with FEM modeling in the Abaqus/CAE software. The normalized spectral plots, obtained from the recorded time histories at receiver 4 for three different stress–strain relationships are shown in Figure 12. The normalized curves are plotted in a logarithmic scale to provide a clear understanding of how sideband peaks change as input excitations increase. Sideband peak amplitude variations as a function of input excitations can be used to determine whether the response is linear or nonlinear [26]. For linear responses, the normalized plots should not show any difference but for nonlinear responses the normalized plots should show some difference when the amplifying factor (AF) is changed. Figure 12a–c correspond to linear, quadratic and square root stress–strain relationships, respectively. As expected, in Figure 12a, the three curves corresponding to AF = 1, 2 and 3 overlap.

**Figure 13:**
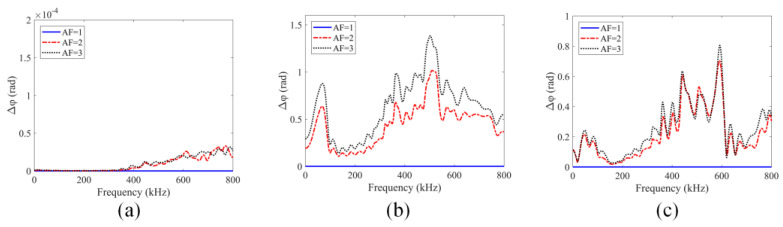
Geometric phase change variations for different stress–strain relationships: (**a**) linear; (**b**) quadratic; (**c**) square root stress–strain relationship.

It can be seen in Figure 12a that in these normalized plots for linear stress–strain relationships both the main peaks (the lobes around the central frequency of 200 kHz) and the sideband peaks (which are further away from the 200 kHz central frequency) show no difference for different AF (amplifying factor) values. For the quadratic stress–strain relationship, the spectral values plotted in Figure 12b show that as the excitation amplitude increases the main lobe amplitudes in the normalized plots do not change but the sideband peak amplitudes decrease. Since, for the quadratic stress–strain relationship, the stiffness of the material increases with load, the higher excitation makes the material harder and produces lower sideband peak amplitude values. Similarly, for square root stress–strain relationships, as can be seen in Figure 12c, the sideband peaks also show nonlinearly—this time, the sideband peak amplitudes increase as the AF increases while the main lobe amplitudes remain unchanged in the normalized plots. The GPC plots for these three types of materials are shown in Figure 13. Figure 13a–c correspond to linear, quadratic and square root stress–strain relationships, respectively.

**Figure 14:**
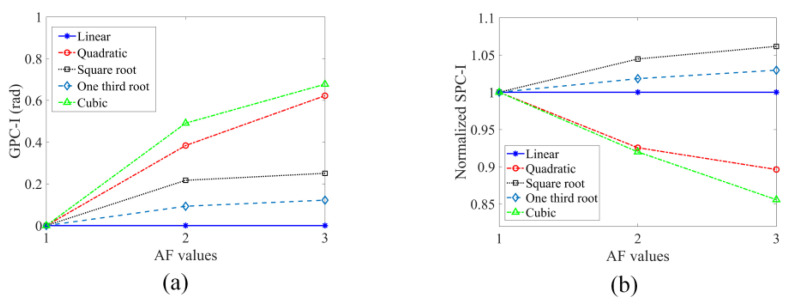
(**a**) Geometric phase change index (GPC-I); (**b**) normalized SPC-I variations for material nonlinearity for different stress–strain relationships.

It can be seen from Figure 13a that for linear stress–strain relationships the GPC plots show no changes as input excitations increase from 0 to around 300 kHz. However, some small non-zero values at higher frequencies from approximately 300 kHz to 800 kHz are observed. Theoretically, for linear stress–strain relationships, there should be no change in the normalized plots when input excitations increase as shown in Figure 12a (the sideband peaks do not show any changes). Those oscillations captured in GPC plots at higher frequencies may arise due to numerical errors since stable conditions cannot be strictly satisfied without significantly reducing the element size when higher frequency waves (having much smaller wavelengths) are modeled. Also, random noise from truncations in FFT may affect the high-frequency behaviors of GPC plots. However, it should be noted that the maximum magnitudes of these oscillations are around 3 × 10^−5^ rad at 800 kHz which is almost zero in comparison to the scale (10^0^) of Figure 13b,c. Therefore, those very small values can be ignored and assumed to be zero for the linear stress–strain relationship. Clear deviations from zero of the GPC plots can be observed for the two nonlinear materials (shown in Figure 13b,c) when input excitations increase. Therefore, GPC can capture material nonlinearity in structures. The GPC-I values for these three types of materials, for which GPC plots are shown in Figure 13 from 0 kHz to 800 kHz, are obtained and plotted in Figure 14a. The normalized SPC-I variations from spectral plots shown in Figure 12 are also obtained and shown in Figure 14b.

It can be seen from the GPC-I plots in Figure 14a that for linear stress–strain relationships GPC-I values do not change as input excitations increase, while for the other two nonlinear materials GPC-I values increase as input excitations increase. For the SPC-I plots shown in Figure 14b, for the linear material, SPC-I values do not change as input excitations increase, which is consistent with what the GPC-I plots show. However, for the other two nonlinear stress–strain relationships—quadratic and square root—the SPC-I plots show two opposite trends for the two types of nonlinearities. When input excitations increase, an increasing trend is observed for the square root stress–strain relationship while a decreasing trend is found for the quadratic stress–strain relationship. Such differences between GPC-I and SPC-I variations for nonlinear materials can be explained in the following manner. GPC-I shows the degree of deviations between the perturbed system (when AF is not equal to 1 in linear and nonlinear materials) and the reference system (AF = 1). Both quadratic and square root stress–strain relationships lead to perturbations from the reference state. The GPC-I senses and plots higher differences for higher AF values. In the SPC-I technique, the variations are related to the sideband peaks’ amplitudes, which are linked to the stiffness of the material. When the stiffness of the material increases with strain (in the quadratic case), the generated nonlinear strain levels for the same stress levels become smaller, thus causing lower sideband peak amplitudes. When the stiffness of the material decreases with strain (in the square root case), the generated strains for the same stress level become larger, thus causing higher sideband peak amplitudes. In addition to the three stress–strain relationships discussed above, two more (one-third root and cubic stress–strain relationships) are analyzed. The SPC-I and GPC-I variations for all five cases are shown in Figure 14. The expected trends are observed: the one-third root relationship is similar to the square root relationship (since here material stiffness decreases as excitation increases), and the cubic relationship is similar to the quadratic relationship (since here material stiffness increases as excitation increases). Both the GPC-I and SPC-I variations could distinctly separate the five different stress–strain behaviors of the material. It can be also concluded from Figure 14 that SPC-I is more effective for distinguishing between different types of material nonlinearity than GPC-I. From the SPC-I plots (noticing whether the variation has an upward or a downward trend) one can say whether the material stiffness increases or decreases when the excitation amplitude increases which is not possible from the GPC-I plots.

## 5. Conclusions

In this work, a numerical investigation is carried out to compare the effects of damage initiation, damage growth and the change in material nonlinearity on the nonlinear ultrasonic technique SPC-I and the TA sensing technique GPC-I. Damage growth (increasing crack thickness) problems are modeled by two numerical techniques—the local finite element method (FEM) and the nonlocal peridynamics (PD)-based peri-ultrasound modeling technique. The effectiveness of the FEM and peri-ultrasound techniques in terms of modeling wave propagation in nonlinear and linear materials in the presence or absence of cracks is also investigated in depth. In FEM modeling, the surface of cracks is not artificially changed. No compressive or shear springs are placed inside the crack, unlike the case in some FE-based models to represent partially closed cracks. Thus, elastic waves cannot pass through an open crack and only purely linear scatterings are generated. However, in nonlocal peri-ultrasound modeling, due to the nonlocal effect, the elastic waves can pass through cracks so both linear and nonlinear responses are generated. A new acoustic parameter called the geometric phase change- index (or GPC-I), which is generated from TA sensing, is defined and adopted to monitor damage growth and material nonlinearity. In damage growth problems, the numerical results show that, for a linear response generated by FEM modeling, the GPC-I is sensitive enough to detect defects but cannot monitor damage growth. In the combined linear and nonlinear responses generated by peri-ultrasound modeling, at the higher frequency range, the GPC-I and SPC-I techniques show consistent trends—first increasing and then decreasing as damage grows. These results indicate that GPC-I can be used to detect the initiation of damage in structures from the linear analysis. From the combined linear/nonlinear analysis, it is shown that GPC-I can also be used effectively to monitor damage growth by selecting only the higher frequencies in the analysis. The higher frequency boundaries are selected from the GPC plots. Recently, using a scanning laser doppler vibrometer (LDV), experimental investigation has been carried out to see if GPC-I and SPC-I can detect and monitor damage growth [43]. In this experimental investigation, the submillimeter indentations served as surrogates for flaws/defects. Similar to the numerical predictions in the current paper, in the experimental paper it was shown that the variations in the geometric phase and the SPC-I in the vicinity of the indentations are significant. The magnitudes of the variations in geometric phase and SPC-I have been shown to be strongly dependent on the number of indentations, i.e., on the level of damage to the plate.

The effect of pure material nonlinearity on GPC (in the absence of any cracks) is investigated by FEM modeling. Different stress–strain relationships—linear and nonlinear—are considered. The numerical results show that for linear materials the GPC-I obtained from the normalized plots does not change as the input excitation increases while for nonlinear materials—with quadratic and square root stress–strain relationships (as well as for cubic and one-third root stress–strain relationships) the GPC-I increases as the input excitations increase, which indicates that nonlinear material behavior can be detected by the GPC-I technique. SPC-I analysis is also carried out for the same nonlinear materials. For linear, square root and one-third root stress–strain relationships, SPC-I shows consistent trends with GPC-I plots. However, for quadratic and cubic stress–strain relationships, SPC-I shows a decreasing trend as input excitations increase, which is the opposite of the GPC-I trends. This is because GPC-I measures the degree of deviations from the reference state (the linear material), and any perturbations introduced into the reference state can result in an increase in GPC-I values. The SPC-I technique, on the other hand, measures the number and strength of sideband peaks generated due to nonlinearity. The material’s nonlinearity causes the stiffness changes with loading. Such stiffness changes affect the amplitudes and the number of sideband peaks. Therefore, SPC-I can capture trends in the change in material stiffness—whether increasing or decreasing with the excitation amplitude. For quadratic and cubic materials, the SPC-I shows a downward trend as load increases, while for square root and one-third root stress–strain relationships, the SPC-I shows an upward trend as load increases. Thus, the SPC-I technique can not only detect material nonlinearity but can also distinguish between types of nonlinearity. From its variation it can identify whether the material stiffness increases or decreases with increasing strain.

## Figures and Tables

**Table 1:** Plate material properties for FEM and peri-ultrasound numerical modeling.

Parameter	Young’s Modulus (GPa)	Poisson’s Ratio	Density (kg/m³)
Values	71.50	0.33	2700.00

## Data Availability

The dataset is available on request from the authors.
